# A new phenotype of anorexia nervosa: the changing shape of eating disorders

**DOI:** 10.1186/2050-2974-1-S1-O44

**Published:** 2013-11-14

**Authors:** Melissa Whitelaw, Heather Gilbertson, Katherine J Lee, Mick B Creati, Susan M Sawyer

**Affiliations:** 1 Department of Nutrition and Food Services, The Royal Children's Hospital, Australia; 2 Centre for Adolescent Health The Royal Children's Hospital, Australia; 3 Department of Paediatrics, The University of Melbourne, Australia; 4 Murdoch Children's Research Institute, Australia

## Background and aims

Adolescents are increasingly referred to our specialist eating disorder (ED) program having lost large amounts of weight and having the diagnostic features of anorexia nervosa (AN) with the exception of underweight. Many of these adolescents with EDNOS-AN[wt] were premorbidly overweight. We aimed to identify the changing prevalence of this phenotype from 2005-2010 in an inpatient sample, and compare the associated complications with adolescents with AN.

## Methods

A 6-year retrospective cohort study was undertaken of adolescents admitted to hospital for the first time for refeeding. All patients met DSM-IV diagnostic criteria for AN (N=72) or EDNOS-AN[wt] (N=28). Baseline medical data are reported.

## Results

In 2005, EDNOS-AN[wt] represented 7.7% of first admissions, rising 6-fold to 46.0% by 2010. Hypophosphataemia (< 1.10mmol/L) developed in 38.9% of AN and 28.6% of EDNOS-AN[wt] inpatients. Bradycardia (pulse rates <50 bpm) occurred in 33.3% of AN and 46.4% of EDNOS-AN[wt] patients, while pulse rates < 40 bpm occurred in 25.0% and 32.0% respectively.

## Conclusions

A dramatic increase in the proportion of adolescents admitted with EDNOS-AN[wt] is evident. The rate of severe medical complications is concerning, and suggests that significant weight loss can be life-threatening in adolescents, even when they are not underweight.

This abstract was presented in the **Anorexia Nervosa – Characteristics and Treatment** stream of the 2013 ANZAED Conference.

